# Presence of bacterial DNA in thrombotic material of patients with myocardial infarction

**DOI:** 10.1038/s41598-020-73011-5

**Published:** 2020-10-01

**Authors:** P. Piñon-Esteban, L. Núñez, R. Moure, G. M. Marrón-Liñares, X. Flores-Rios, G. Aldama-Lopez, J. Salgado-Fernandez, R. Calviño-Santos, F. Rebollal-Leal, R. Pan-Lizcano, N. Vazquez-Gonzalez, G. Bou, M. Tomás, M. Hermida-Prieto, J. M. Vazquez-Rodriguez

**Affiliations:** 1 Servicio de Cardiología. Complexo Hospitalario Universitario de A Coruña (CHUAC), Sergas, Instituto de Investigación Biomédica de A Coruña (INIBIC), Universidade da Coruña(UDC), As Xubias 84, 15006 A Coruña, Spain; 2 Grupo de investigación en Cardiología, Instituto de Investigación Biomédica de A Coruña (INIBIC), Complexo Hospitalario Universitario de A Coruña (CHUAC), Sergas. Universidade da Coruña (UDC), 15006 A Coruña, Spain; 3 Grupo de investigación en Microbiología, Instituto de Investigación Biomédica de A Coruña (INIBIC)Complexo Hospitalario Universitario de A Coruña (CHUAC)Sergas. Universidade da Coruña (UDC), 15006 A Coruña, Spain; 4 CIBERCV (Centro de Investigación Biomédica en Red Enfermedades Cardiovasculares), Instituto de Salud Carlos III, Madrid, Spain

**Keywords:** Biological techniques, Cardiology, Medical research

## Abstract

Infectious agents have been suggested to be involved in etiopathogenesis of Acute Coronary Syndrome (ACS). However, the relationship between bacterial infection and acute myocardial infarction (AMI) has not yet been completely clarified. The objective of this study is to detect bacterial DNA in thrombotic material of patients with ACS with ST-segment elevation (STEMI) treated with Primary Percutaneous Coronary Intervention (PPCI). We studied 109 consecutive patients with STEMI, who underwent thrombus aspiration and arterial peripheral blood sampling. Testing for bacterial DNA was performed by probe-based real-time Polymerase Chain Reaction (PCR). 12 probes and primers were used for the detection of *Aggregatibacter* *actinomycetemcomitans*, *Chlamydia* *pneumoniae*, *viridans group streptococci*, *Porphyromonas* *gingivalis*, *Fusobacterium* *nucleatum*, *Tannarella* *forsythia*, *Treponema* *denticola*, *Helycobacter* *pylori*, *Mycoplasma* *pneumoniae*, *Staphylococus* *aureus*,  *Prevotella intermedia* and *Streptococcus* *mutans*. Thus, DNA of four species of bacteria was detected in 10 of the 109 patients studied. The most frequent species was *viridans group streptococci* (6 patients, 5.5%), followed by *Staphylococus* *aureus* (2 patients, 1.8%). Moreover, a patient had DNA of *Porphyromonas* *gingivalis* (0.9%); and another patient had DNA of *Prevotella intermedia* (0.9%). Bacterial DNA was not detected in peripheral blood of any of our patients. In conclusion, DNA of four species of endodontic and periodontal bacteria was detected in thrombotic material of 10 STEMI patients. Bacterial DNA was not detected in the peripheral blood of patients with bacterial DNA in their thrombotic material. Bacteria could be latently present in plaques and might play a role in plaque instability and thrombus formation leading to ACS.

## Introduction

Infectious agents have been suggested to play a role in chronic inflammation, which is involved in the etiopathogenesis of atherosclerosis^1^. The infection/atherosclerosis paradigm has been studied since the 1970s. A number of findings associate pathogens with atherosclerosis^2^, namely: (a) pathogens have been found in human atherosclerotic vessels^3^; (b) infection has been reported to lead to atherosclerosis^4^ in animal studies; (c) seroepidemiological studies have demonstrated an association between pathogen- specific antibodies and atherosclerosis^5^. Multiple pathogens have been documented to be involved in these associations, including species of bacteria such as *Chlamydia* *pneumoniae*, *Helicobacter* *pylori*, and periodontal pathogens^1^.

Of these infectious agents, the pathogen which association with atherosclerosis has been most extensively studied is *Chlamydia* *pneumoniae*, with different results. *Chlamydia* *pneumoniae* was the first infectious organism to be found in cells of human atherosclerotic plaques, but rarely in normal arterial cells. It is also one of the few agents detected in plaques from which viable organisms have been isolated^3,6–10^. However, antibiotic treatment against *Chlamydia* *pneumoniae* has shown no benefit in secondary prevention of coronary events^11^. *Helicobacter* species have also been found by Polymerase Chain Reaction (PCR) in biopsy specimens from coronary plaques of patients who underwent coronary artery bypass grafting^12^.

Regarding oral bacteria, a large number of infectious agents have been associated with an increased risk for cardiovascular disease. Periodontal bacteria have been detected in atherosclerotic plaques in coronary arteries by PCR^13,14^. Periodontitis, a chronic tissue-destructive inflammatory disease predominantly caused by Gramm-negative bacteria, has been associated with a higher risk for acute myocardial infarction^15^ (AMI) and coronary heart disease^16^. An obvious possibility is that these diseases are promoted by shared risk factors. Yet, periodontitis has been postulated—but not confirmed—to cause cardiovascular disease.

Two recent studies have demonstrated the presence of DNA of periodontal and endodontic bacteria in thrombotic material of patients with Acute Coronary Syndrome with ST-segment Elevation (STEMI) treated with Primary Percutaneous Coronary Intervention (PPCI). In one of these studies, Ohki et al^17^ included 81 patients and analyzed the presence of five periodontal bacteria, of which they detected three species: *Aggregatibacter* *actinomycetemcomitans*, *Porphyromonas* *gingivalis* and *Treponema* *denticola*. In another study, Pessi et al.^18^ confirmed the presence of DNA of bacteria typically involved in endodontic infection, mainly oral *viridans streptococci*, and of periodontal pathogens in thrombotic material of 101 STEMI patients.

Hence, there is no agreement about the association between some microorganisms and AMI. The aim of our study is to investigate the presence of DNA of bacteria typically involved in chronic inflammation such as *Chlamydia* *pneumoniae*, *Helicobacter* *pylori*, and *Mycoplasma* *pneumoniae*, and of periodontal and endodontic bacteria in thrombotic material and peripheral blood of STEMI patients treated with Primary Percutaneous Coronary Intervention in a reference hospital in Spain.

## Methods

### Patients inclusion

The sample of this study was composed of 175 consecutive patients with STEMI treated with PPCI in which intracoronary thrombus was obtained through catheter aspiration, in A Coruña University Hospital, Spain, between March 2013 and January 2016. Thrombi were removed by aspiration and arterial peripheral blood was sampled. Aspiration of thrombi from the culprit artery using sterile equipment (Export Catheter, Medtronic) was part of the standard management of AMI in our center. Following aspiration, the aspirate was poured into a pyrogen-free Eppendorf tube. In total, 5 mL of blood was extracted via the arterial sheath and subsequently stored. The thrombotic material was immediately stored in liquid Nitrogen. Blood samples were frozen at – 80 °C until analysis. Percutaneous coronary intervention was performed according to local standards. Aspirate sampling did not affect patients’ treatment. The study was approved by the "*Comité* *ético* *de* *investigación* *de* *Galicia*" (Reference: 2012/222) and conforms to the ethical standards of the Declaration of Helsinki. Written informed consent to aspirate sampling and DNA testing was obtained. Samples were registered in the biobank of samples for research on hemodynamics of the National Biobank Network of Carlos III Health Institute (C.0002483).

Clinical data including the presence of major coronary risk factors, severity of coronary artery disease, location of AMI, and prognosis during follow-up were recorded to compare groups according the presence of bacterial DNA (positivity/negativity, DNA [+] and [−] groups).

### DNA extraction

DNA was extracted from frozen thrombotic material using the Illustra DNA Extraction Kit BACC3 (GE Healthcare) following the protocol, with minor modifications. Mechanical tissue disaggregation was specifically designed for preparing thrombotic material for DNA extraction. DNA purity and concentration were measured using a NanoDrop spectrophotometer. The minimum quality (ng/µL) and purity values (ratio 260/280) accepted for qPCR analysis were 5 and 1.5, respectively. Genomic DNA was isolated from peripheral blood with Gentra Puregene Blood Kit (Qiagen), as previously described^19,20^, only in patients with a bacterial DNA-positive thrombus (DNA [+]).

### Bacterial DNA detection Primers and probe design

A collection of primers and a set of UPL short probes (Roche Molecular Systems Inc., Pleasanton, CA, USA) were designed in order to detect the oral pathogens most frequently found in coronary thrombotic material (Table 1). A positive Beta globin control was used to verify the validity of the extraction and amplification procedure (primers described in Table 1). Likewise, a negative control including distilled water instead of the DNA template was used to verify the absence of reagent contamination.

**Table 1:** Species specific primers and probes to detect ADN from different bacterias.

Oral pathogens	Not oral pathogens
*viridans* *group* *Streptococci* Fw: TCCGTAAACAATTGGACGAA Rv: CACGTTGGATACCACGAAGA Probe: 67 UPS	*Helicobacter* *pylori* Fw: CCAATGGTAAATTAGTTCCTGGTG Rv: GGTCTGTCGCCAACATTTTT Probe: 155 UPS
*Streptococcus* *mutans* Fw: GGGTTCTAAGCGCCACATT Rv: CGAACTAGTACTGTTGTGGCTAGG Probe: 59 UPS	*Chlamydia* *pneumoniae* Fw: CGTTGGCTACGAAAGATTCAG Rv: GGGTAAAGAATAACGAGCACCA Probe: 67 UPS
*Staphylococcus* *aureus* Fw: CCTTTTGTAGACTCTTCGTCAAATTC Rv: CATCACGCTGATGTTGTTGA Probe: 49 UPS	*Micoplasma* *pneumoniae* Fw: GCAGCTAATGTCCGTAGTGCT Rv: GTGGTGAGCATGACCAGGTT Probe: 150 UPS
*Porphyromonas* *gingivalis* Fw: ATAGTAGCGTGTCCGGCTTC Rv: ATCGTAGGCGGATTGGAGA Probe: 82 UPS	
*Fusobacterium* *nucleatum* Fw: TCCCAGCAAATGTTGGAAG Rv: TTCATCATCAAATTCGTCATAGTCT Probe: 143 UPS	
*Treponema* *denticola* Fw: GCAGATATACAGGTAGACATAGGAAGC Rv: GCTCTTCTAAGTCCTGTTCAGTGTT Probe: 128 UPS	
*Prevotella intermedia* Fw: TGGTATCAAAATCAGCAAGGAA Rv: CGTCTTCGCGCATATTCAG Probe: 126 UPS	
*Tannerella* *Forsythia* Fw: AAACATCGTGGATACCCTCCT Rv: ATCAAAGTGGTCGGTGTCG Probe: 70 UPS	
*Aggregatibacter actinomycetemcomitans* Fw: CAATACTACGGTGGTGCAGTATCT Rv: ATATTGTTGGCGCGTAATGC Probe: 67 UPS	
**Positive control**	
*Beta* *globin* Fw: CAAATAAAGGACAGTGCAGAAATC Rv: CTCCTTCTGCCCTTCTAGGC Probe: 144 UPS	

*Fw* forward, *Rv* reverse, *UPS* universal probe library.

### Real-time PCR

Thermal cycling was carried out using the LightCycler 480 II system (Roche Molecular Systems Inc.). Each reaction was prepared for singleplex assay—a pathogen *per* reaction—by mixing 5 µL of the DNA template with 10 µL of master mix (LightCycler 480 Probes Master; Roche Molecular Systems Inc.); 2.8 µL of RNase free distilled water; 0.75 µL of primers (forward and reverse; 20 µM each), 0.5 µL of the UPL probe; and 0.2 µL of Uracyl N-glycosilase (Arcticzymes AS, Norway) to minimize the risk of contamination due to persistent templates. The mixture was dispensed in a 96-well microtiter plate sealed with foil (Roche Molecular Systems Inc.) for 5-min incubation at room temperature for UNG activation. The real-time PCR protocol consisted of initial preincubation for enzyme activation (Fast-start) at 95 °C for 10 min, and 45 cycles of denaturation at 95 °C for 10 s, 60 °C for 20 s for annealing, and 1 s of elongation at 72 °C, followed by a cooling hold of 10 s at 40 °C. The LightCycler 480 II system could yield three possible results for target detection, namely: positive when a signal was detected at a Ct < 40; negative if no signal was detected at Ct > 45; and an indeterminate result if a signal was detected at a late Ct (40 < Ct < 45).

### Statistical analysis

SPSS version 19.0 was used for statistical analysis (IBM SPSS Statistics, Chicago, IL, USA). Continuous variables are presented either as mean (Standard Deviation [SD]) or as median values (interquartile range [IQR]). Categorical variables are expressed as absolute and relative frequencies (percentages). Associations between the presence of bacterial DNA (positivity/negativity) in the sample and clinical and angiographic parameters were calculated using Fisher’s exact test. To calculate event-free survival, Kaplan Meier curves and log Rank Tests were used. Mann–Whitney *U* Test was used for comparison of non-parametric numerical variables. A value of *p* < 0.05 was considered statistically significant.

## Results

Samples from 175 patients were analyzed. A total of 66 patients were excluded, as the DNA extracted from thrombus aspirates did not reach the quality and purity standards established. Finally, we analyzed the samples of 109 patients, who constituted the study group. Baseline characteristics of study population are shown in Table 2. The 109 patients with STEMI received antithrombotic therapy with Acetilsalicic Acid, Clopidogrel, Ticagrelor or Prasugrel, according to the local AMI management protocol.

**Table 2:** Baseline Characteristics of study population.

Baseline characteristics	N = 109
Age (years mean ± SD)	61.4 ± 14.1
Male (%)	83 (76.1%)
Smokers (%)	41 (37.6%)
Hypertension (%)	47 (43%)
Dyslipidaemia (%)	36 (33%)
Diabetes mellitus (%)	14 (12.8%)
Prior MI (%)	6 (5.5%)
Anterior MI (%)	38 (35%)
Inferior MI (%)	52 (47.7%)
Radial access (%)	93 (85%)
Multivessel disease	39 (35.7%)
**Infarct related artery**	
LAD (%)	46%
LCx (%)	12%
RCA (%)	39%
Success procedure (TIMI III)	94%

*MI* myocardial infarction, *LAD* left anterior descending artery, *LCx* left circumflex, *RCA* right coronary artery, *SD* Standard Deviation

### Presence of bacterial DNA in thrombi from AMI patients

Of the 10 microorganisms tested, DNA of four different species of bacteria was detected in 10 thrombus samples (9.2%) of the 109 patients studied. The bacterial DNA most frequently detected corresponded to *viridans* *group* *streptococci*, which was identified in six of the 109 patients examined (5.5%). The species of some bacteria could not be identified due to the low DNA concentration extracted. Yet, we are certain that the species that could not be identified did not correspond to *Streptoccus* *mutans*, since we used probes and primers for the amplification of this species. Two typical species of microbiota in periodontal disease, *Porphyromonas* *gingivalis* and *Prevotella intermedia*, were identified in thrombotic material of two patients. Moreover, DNA from *Staphyloccus* *aureus* was detected in two patients (1.8%). We did not find bacterial DNA in peripheral blood of patients with bacterial DNA in their thrombotic material.

Regarding the association between bacterial DNA and clinical and angiographic characteristics, no differences were found based on age, sex, infarct location, number of vessels diseased, presence of diabetes mellitus or other risk factors (Table 3). Two patients had recent exposure to antibiotics; one of them, the patient in whom DNA of Porphiromonas gingivalis was detected in the intracoronary thrombus, was receiving treatment with Amoxicillin for an infection of the upper respiratory tract; the other patient was receiving Norfloxacin treatment and no bacterial DNA was found in this patient.

**Table 3:** Differences between DNA ( +) and DNA (-) groups.

Baseline characteristics	No bacterial DNA in thrombotic material	Bacterial DNA in thrombotic material	*p*
	n = 99	n = 10	
Age (years mean ± SD)	61.4 ± 14.2	61.6 ± 13.0	0.97
Male (%)	75 (75.8%)	8 (80.0%)	0.55
Smokers (%)	37 (37.4%)	4 (40.0%)	0.56
Hypertension (%)	43 (43.4%)	4 (40.0%)	0.55
Dyslipidaemia (%)	34 (34.3%)	2 (20.0%)	0.29
Diabetes mellitus (%)	11 (11.1%)	3 (30.0%)	0.11
Prior MI (%)	5 (5.1%)	1 (10%)	0.44
Anterior MI (%)	45 (48%)	4 (40%)	1.0
Inferior MI (%)	48 (52%)	4 (40%)	1.0
Radial access (%)	86 (86.9%)	7 (70%)	0.16
Multivessel disease	39 (36.4%)	4 (40%)	0.53
**Infarct related artery**			
LAD (%)	47 (47%)	4(40%)	0.74
LCx (%)	12 (12.1%)	1 (10%)	1.0
RCA (%)	38 (38%)	5 (50%)	0.512
**Success procedure (TIMI III)**			
	92 (93%)	9 (90%)	0.50

Associations between the presence of bacterial DNA (positivity/negativity) in the sample and clinical and angiographic parameters were calculated using Fisher’s exact test. Mann–Whitney U Test was used for comparison of non-parametric numerical variables. A value of p < 0.05 was considered statistically significant.

*MI* myocardial infarction, *LAD* left anterior descending artery, *LCx* left circumflex, *RCA* right coronary artery.

The median follow up was 420 days and no significant differences in outcomes were observed between DNA [+] and DNA [−] patients in terms of events during follow-up; during this period there were six deaths (6.1%) in the DNA [−] group and one death (10%) in the DNA [ +] group; three non-fatal AMI (3.1%) in the DNA [−] group and one non-fatal AMI in the DNA [+] group.

## Discussion

Our results confirm the presence of bacterial DNA in the thrombotic material of patients with AMI. In the present study, 9.2% of thrombus samples contained bacterial DNA, mainly from *viridans* *group* *streptococci*, which are typically involved in endodontic infection. Notably, we detected DNA of other endodontic bacteria—such as *Staphylococcus* *aureus*—and periodontal pathogens—as *Porphyromonas* *gingivalis* or *Prevotella* *intermedia*. In our study, the peripheral blood of patients was only analyzed when their thrombus was positive for bacterial DNA. It is important to note that the absence of bacterial DNA in peripheral arterial blood automatically excluded the presence of active clinically-silent bacteriemia at the moment of MI. Besides this, potential contaminations during sampling can be confidently excluded, since thrombotic material and peripheral blood were collected using the same procedure.

Our results are consistent with those reported in two recent studies in patients with AMI conducted in Japan and Finland. Ohki et al.^17^ analyzed thrombotic material of 81 patients with AMI treated with PPCI. The authors investigated the presence of five periodontal bacteria by PCR and detected three species, namely: *Aggregatibacter* *actinomycetemcomitans*, *Porphyromonas* *gingivalis* and *Treponema* *denticola* in 18 patients (22.2%). Pessi et al.^18^ analyzed thrombotic material and arterial blood of 101 AMI patients undergoing PPCI by real-time quantitative PCR. The authors investigated the presence of oral bacteria and DNA of *Chlamydia* *pneumoniae*. In this study, DNA of bacteria generally found in endodontic infection—mainly oral *viridans* *group* *streptococci*—was detected in 78.2% of samples, whereas periodontal pathogens were detected in 34.7%. In agreement with our results, Pessi et al. did not find DNA of *Chlamydia* *pneumoniae* either. Therefore, DNA of similar oral pathogens was found in thrombotic material in the three studies, whereas DNA of bacteria traditionally related to the etiopathogenesis of atherosclerosis was not detected.

Although numerous seroepidemiological, histopathological, and animal studies have associated *Chlamydia* *pneumoniae* with atherosclerosis, we did not find DNA of *Chlamydia* *pneumoniae* in our patients. *Chlamydia* *pneumoniae* is the species of bacteria linked to atherosclerosis that has been most widely studied. Several techniques have been used to detect the presence of this species of bacteria in vascular tissues^21^, including PCR, electron microscopy, and immunocytochemistry. However, the results of previous studies vary greatly, and there are no reports of the presence of *Chlamydia* *pneumoniae* in thrombotic material of patients with AMI. We did not find other microorganisms reportedly associated with atherosclerosis, as *Helycobacter* *pylori* and *Mycoplasma* *pneumoniae.*

Numerous studies have related oral disease and cardiovascular events. The relationship between periodontitis and cardiovascular disease was assessed in the PAROKRANK STUDY^15^. This case–control study including 805 patients with a first episode of AMI revealed after adjustment for confounding factors that the risk for this first episode of AMI was significantly higher in patients with periodontitis.

Differences in the percentage of samples with bacterial DNA between our study and previous studies could be explained by several reasons. The first reason is methodological, as one of the studies sampled intracoronary thrombus and 2 ml of intracoronary blood samples^17^, whereas we sampled only intracoronary thrombotic material. If we had collected coronary blood as did Okhi et al., the detection rate might have been higher to the one obtained. Another factor that could explain variability of results is epidemiological, with differences in dental health and incidence of periodontal disease between study populations. In the study published by Pessi et al. 30 patients (29.5%) were referred for a dental panoramic tomography. These tomographies showed a periapical abscess in 46.6% of patients. A significant association was observed between periapical abscess confirmed by tomography and the detection of *streptococcus mitis* group in thrombotic material.

In our study, seven of the ten patients with DNA (+) thrombus material had a history of tooth extraction. The reason of extraction was periodontitis in two patients, and unknown in the others. Examining differences in the incidence of periodontal infection between the DNA (+) and DNA (−) groups would have been of interest.

There were no differences between groups regarding their clinical characteristics. We observed a non-significant trend towards a higher prevalence of Diabetes Mellitus in the DNA (+) group, which is a well-known risk factor for dental infection. However, the small sample limits the statistical power of analyses. Although some reports suggest a relationship between severity of coronary disease and dental infection^22^, no differences were observed in our study between the DNA (+) and DNA (−) group regarding the severity or extent of coronary artery disease or other clinical characteristics. These results are consistent with the ones of the studies published by Okhi et al. and Pessi et al.

In relation to prognosis, no differences were documented in outcomes based on the presence or not of bacterial DNA in thrombotic material. Thus, there were no differences between groups in mortality rates, occurrence of a new AMI, or stent thrombosis during follow-up. However, these results are influenced by the small size of the sample and low number of events.

There are around 1000 different species in the oral cavity, which are prevailingly *streptoccci*^23^. *Viridans* *streptococcus* is a pseudotaxonomic non-Linnean term for a group of human commensals most commonly present in the oral cavity. These bacteria have several properties that indicate that, rather than being an innocent bystander in atherosclerosis, they could play an active role in the development of the disease. Such bacteria would be latently present in plaques and be involved in plaque instability, which leads to subsequent thrombus formation and cause Acute Coronary Syndrome. Whether these bacteria have a role in the atherosclerotic plaque rupture associated with acute coronary syndromes remain to be determined. Also, it is unknown whether these bacteria promote the atherosclerotic plaque or attach to existing atherosclerotic lesions. It has been suggested that these microorganisms trigger the production of inflammatory cytokines, monocyte chemoattractant proteins^24^, and the accumulation of macrophages by the activation of the CD14/Toll-like receptor 2 complex^25^. They are able to attach to different surfaces and generate a biofilm, thereby enabling other bacteria to infitrate tissue^26^. These microorganisms also have thrombogenic properties with potential to initiate or contribute to platelet aggregation^27^. *Porphyromonas* *gingivalis* (PG) has been reported to hydrolyze a fibrous cap and, as *viridans* *streptococci,* it can induce platelet aggregation^28^. After plaque rupture, bacteria may be released into the blood stream and induce platelet aggregation. Also, these bacteria can be detected in thrombotic material as demonstrated by Pessi et al., who found the bacteria in 3 of the 9 thrombotic material samples detected by transmission electron microscopy. The route by which these microorganisms reach atherosclerotic lesions remains unclear. Bacteriemia originated in the oral cavity is the most widely accepted theory. After oral manipulation, like tooth extraction or simply tooth brushing, the patient can develop transient bacteriemia, which is usually subclinical and mostly caused by *viridans* *streptococci*^29,30^. Several studies have shown that patients with periodontitis are at a higher risk for bacteriemia^31,32^, and some living microorganisms may reach coronary lesions. Bacteria may reach atherosclerotic lesions directly through the endothelium and be phagocytosed by T-cells and finally translocated into an atherosclerotic plaque. Numerous repeated bacteriemias or other bacterial infections during lifetime would contribute to accumulate pathogens in atherosclerotic lesions, thereby contributing to the development of chronic low-grade inflammation. T-cell mediated release of proinflammatory mediators and proteolytic substances in response to a chronic low- grade bacterial infection may be the mechanism by which thrombosis is induced. In our study, we only investigated the presence of bacterial DNA in thrombotic material, but we did not find bacterial DNA in peripheral blood. This finding could suggest the presence of a local mechanism of action where bacteria could play a role in the development of acute coronary syndromes. Thus, the DNA detected may correspond to living bacteria present in the coronary atheroma, rather than from phagocytic cells from circulation.

## Limitations

The present study has several limitations. Firstly, not all thrombi could be studied, as 66 of the 175 samples were excluded. In addition, the amount of material obtained from some patients was limited. Secondly, due the limited sampled obtained by aspiration, we were not able to identify species level 6 microorganisms of the *viridans* *group* *streptococci*. Finally, as previously mentioned, we could not collect comprehensive clinical details regarding oral disease in our population, which would have provided relevant information.

## Conclusion

To the best of our knowledge, this is the largest observational study ever performed on the relationship between the presence of DNA of endodontic and periodontal microorganisms in thrombotic material in myocardial infarction. DNA of four species of endodontic and periodontal bacteria was detected in the thrombotic material of 10 STEMI patients. Bacterial DNA was not detected in the peripheral blood of patients with bacterial DNA in their thrombotic material. This finding suggests that endodontic and periodontal bacteria could be latently present in plaques and might play a role in plaque instability and subsequent thrombus formation in a subset of patients, ultimately leading to ACS. This is a pilot study. The role of bacterial infection in coronary events remains unclear. More research is needed to determine the relationship between oral infection and acute coronary events, and assess the potential preventive role of dental care in myocardial infarction.

## Data Availability

The data underlying this article will be shared on reasonable request to the corresponding author.
